# Exogenous gonadotropins do not increase the blood-follicular transportation capacity of extra-ovarian hormones such as prolactin and cortisol

**DOI:** 10.1186/1477-7827-11-87

**Published:** 2013-09-04

**Authors:** Michael von Wolff, Sophie Schneider, Zahraa Kollmann, Benedicte Weiss, Nick A Bersinger

**Affiliations:** 1 University Women’s hospital, Division of Gynaecological Endocrinology and Reproductive Medicine, Effingerstrasse 102, Berne 3010, Switzerland

**Keywords:** Follicular fluid, Natural Cycle In vitro Fertilisation, Permeability, Gonadotropin, Prolactin, Cortisol

## Abstract

**Backgrounds:**

In vitro fertilization involves high dosage gonadotropin stimulation, which apparently has some negative impact on follicular endocrine function. As chorionic gonadotropin stimulation has been shown to increase the blood-follicular permeability in animal models, this raises the question if such an effect also applies to gonadotropins in humans, possibly affecting the endocrine follicular milieu.

**Findings:**

Follicular fluid and serum were collected at the time of follicular aspiration in *in vitro* fertilisation without (Natural cycle IVF, n = 24) and with (conventional gonadotropin stimulated IVF, n = 31) gonadotropin stimulation. The concentration of the extra-ovarian hormones prolactin and cortisol were analysed by immunoassays.

**Results:**

Median serum prolactin and cortisol concentrations were 12.3 ng/mL and 399 nmol/L without *versus* 32.2 ng/mL and 623 nmol/L with gonadotropin stimulation. The corresponding concentrations in follicular fluid were 20.6 ng/mL and 445 nmol/L *versus* 28.8 ng/ml and 456 nmol/L for prolactin and cortisol. As a consequence, mean follicular fluid:serum ratios were significantly reduced under gonadotropin stimulation (prolactin p = 0.0138, cortisol p = 0.0001). As an enhanced blood-follicular permeability and transportation, induced by gonadotropin stimulation, would result in increased instead of decreased follicular fluid:serum ratios as found in this study, it can be assumed that this does not affect extra-ovarian protein and steroid hormones as illustrated by prolactin and cortisol.

**Conclusions:**

The model of serum follicular fluid:serum ratio of hormones, produced outside the ovaries, did not reveal a gonadotropin induced increased blood-follicular transportation capacity. Therefore it can be assumed that the effect of gonadotropins on follicular endocrine function is not due to an increased ovarian permeability of extra-ovarian hormones.

## Findings

### Background

Ovarian follicles are regulated by different hormones acting directly or indirectly on theca cells outside or on granulosa cells inside the follicle. Unaffected transportation of these hormones into the follicle is highly relevant as a decreased hormone concentration in follicular fluid (FF) might negatively influence follicular function. Accordingly, transportation deficits as a source of follicular dysfunction have been suggested.

In conventional In vitro Fertilization (cIVF), high dosages of gonadotropins are used to induce a multifollicular reaction. Gonadotropin stimulation has also many effects on follicular function such as changes in the metabolism of the follicles, possibly being one of the reasons for the suggested lower implantation rates of embryos in cIVF in comparison with those in Natural Cycle-IVF (NC-IVF) without any exogenous gonadotropin stimulation [1,2]. Furthermore, animal studies have shown that chorionic gonadotropin stimulation reduced the resistance of blood-ovarian barrier and thereby increased the influx of microspheres and molecules into the follicles [3,4]. These studies raised the question whether such an effect also applies to gonadotropin stimulation resulting in an increase of the concentration of follicle regulating hormones such as prolactin and cortisol.

We therefore analysed serum (S) and FF concentrations of prolactin and cortisol in cIVF-follicles, aspirated after hCG triggering and calculated the FF:S ratio. As a reference model we used follicles from NC-IVF-therapies. We chose prolactin and cortisol, as both these hormones are not (cortisol) or hardly (prolactin) produced by the follicles and as they have been shown to be relevant for follicular regulation.

### Methods

Fifty-five women underwent either cIVF (n = 31) or NC-IVF (n = 24) according to their personal preference. For cIVF, the antagonist protocol was used. The study was approved by the local ethical committee and patient’s approval was given by written consent. HMG (150 to 300 IU per day of human menopausal gonadotropin, Menogon HP®, Ferring AG, Baar, Switzerland) treatment was initiated between day 3–5 of the menstrual cycle. GnRH antagonists (Orgalutran®, MSD Merck Sharp & Dohme GmbH, Lucerne, Switzerland) were first administered between day 6–7 of the menstrual cycle and continued until ovulation induction. Once an adaequate ovarian response had been confirmed, urinary human chorionic gonadotropin (hCG) (Predalon®, MSD Merck Sharp & Dohme GmbH, Lucerne, Switzerland) were administered to induce ovulation 36 hours before oocyte retrieval.

NC-IVF patients were monitored by ultrasound and analysis of luteinizing hormone and 17β-estradiol (E_2_) concentrations. When the follicle diameter reached at least 18 mm and E_2_-concentration was above 800 pmol/L, hCG were administered 36 hours before oocyte retrieval. Follicle aspirations in both cIVF and NC-IVF were scheduled in the morning to avoid variations of cortisol concentrations due to its circadian release pattern.

The *cumulus oophorus* complex was isolated and the FF clarified by centrifugation, and stored at −30°C until further analysis. Venous blood was collected at the time of follicle aspiration and serum was stored at −30°C.

Prolactin and cortisol concentrations were analysed by electrochemiluminescent immunoassay (ECLIA) on a COBAS 6000 (e601Modul) (Roche Diagnostics GmbH, Mannheim, Germany). The interassay variations of the assays were less than 4%.

Statistical analyses were performed by Mann–Whitney U test for comparisons of hormone levels between the two patient groups, and by Student t-test for the comparisons of the FF:Serum ratios as these latter were found to be normally distributed.

### Results

Mean patient age was 34.9 (SD 4.9, range 21–42) years for the NC-IVF and 33.8 (SD 3.7, range 25–41) years for the cIVF group. Concentration of anti mullerian hormone (AMH) was 17.7 (SD 14.6) and 22.9 (SD 15.3) and mean follicle size at the time of aspiration was 19.6 (SD 1.2) and 19.5 (SD 1.2) mm, respectively. Body mass index in all patients was between 20 and 30.

**Figure 1:**
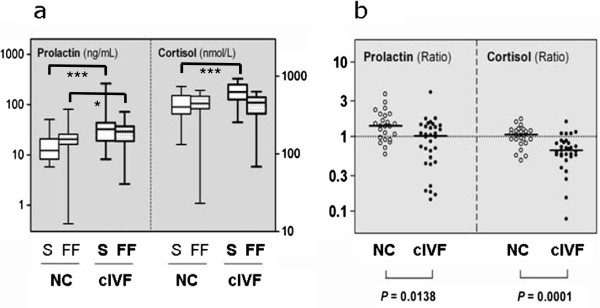
**Prolactin and cortisol in serum and follicular fluid in cIVF and NC-IVF. (a)**. Box-and-whisker plot of prolactin and cortisol concentrations in natural IVF (NC, fine lines) and conventional, gonadotropin stimulated IVF (cIVF, bold lines) cycles in serum (S, open boxes) and in follicular fluid (FF, hatched boxes). Boxes represent the 25th, 50th (median), and 75th centiles, the whiskers indicate the minimum and maximum levels. **(b)**. Scatterplot of individual FF:S ratios in conventional, gonadotropin stimulated IVF (cIVF, closed circles) compared to natural IVF (NC, open circles). The solid horizontal lines indicate the medians. Please note the logarithmic scale in both panels **(a)** and **(b)**.

Median serum prolactin concentrations were 12.3 (range 5.8–50.7) ng/mL in the NC-IVF and around 3-fold higher (median 32.2, range 8.3–261.6 ng/ml) in the cIVF group (p = 0.0002). The same pattern was observed for cortisol; in NC-IVF the serum concentrations were 399 (range 132–735) nmol/L and in cIVF they were higher (median 623, range 253–928 nmol/L (p = 0.0001). In the FF, the increased concentrations of prolactin and cortisol in the cIVF over the NC group were less pronounced (and statistically significant only for prolactin) when compared to the observations in serum. Median FF prolactin levels were 20.6 (range 0.4–80.5) ng/mL in NC-IVF and 28.8 (range 2.7–71.1) ng/mL in cIVF (p = 0.0164), and median FF cortisol concentrations were 445 (range 23–657) nmol/L in NC-IVF and 456 (69–631) nmol/L in cIVF. Serum and FF prolactin and cortisol concentrations are shown as box-and-whisker plots in Figure 1a.

The average FF:S ratios were significantly lower in cIVF than in NC-IVF for both hormones. For prolactin, the mean FF:S ratio was 1.5 (SD, 0.8) in NC-IVF and 1.0 (SD, 0.7) in cIVF (p = 0.0138). For cortisol, the mean FF:S ratios were 1.0 (SD, 0.3) and 0.7 (SD, 0.3) for NC-IVF and cIVF, respectively (p = 0.0001). These results are illustrated as scatter plots in Figure 1b.

### Discussion

Gonadotropins have been associated with several effects on follicular function. Recent research comparing the protein contents of follicles following conventional gonadotropin stimulation in IVF compared with naturally matured follicles has shown differences in the endocrine milieu. In conventional IVF androgens as well as AMH, which has been shown to be a marker of oocyte quality have been found to be much lower concentrated in follicular fluids at the time of follicle aspiration [5,6].

As gonadotropins seem to have a substantial effect on the follicular endocrine milieu, it is of interest, if this effect is also based on an effect of gonadotropins on the blood-follicular transportation capacity and permeability.

In mice [3] and in rats [4] chorionic gonadotropins induced an increase of molecular blood-ovarian permeability and transportation capacity by raising ovarian blood flow and increasing ovarian permeability. This increase in permeability is modulated mainly through increased numbers of large capillary pores, similar to a classical inflammatory response [4]. Furthermore, it seems to be charge and size specific as in mice negatively charged inter-α-inhibitor entered the follicle only after preovulatory stimulation by chorionic gonadotropins and passage of intermediate-size molecules was also chorionic gonadotropin dependant [3].

These findings are limited to animal studies and to stimulation with chorionic gonadotropins but do not involve gonadotropins such as follicle stimulating hormone (FSH) or human menopausal gonadotropin (HMG) as used in IVF therapies. Furthermore, in humans such investigations are difficult to perform and any results will only apply to the specific molecules involved in the analysis. Therefore, studies should directly involve substances of interest which play a role in ovarian function and which are not or hardly produced by the follicles. These criteria apply to prolactin [7], produced by the pituitary gland and to cortisol [8], produced by the adrenal cortex.

We chose the FF:S ratio as a parameter for the serum-ovarian transportation capacity and speculated that this parameter would be higher if the permeability increased due to gonadotropins. Our study revealed exactly the opposite. The FF:S ratio was lower in gonadotropin stimulated cIVF than in NC-IVF patients.

These surprising results might theoretically be due to the following effects: First, the conversion of the studied molecules is activated by gonadotropin stimulation leading to lower FF concentrations; secondly, the result is due to increased serum concentrations, altering the ratios and thirdly the blood ovarian transportation capacity is not increased but decreased by gonadotropins.

For prolactin, no specific enzymes are known which could convert this molecule, resulting in an accelerated gonadotropin induced degradation of the molecule. In contrast, for cortisol an enzyme which converts cortisol in cortisone is known. The enzyme 11betaHSD, expressed by granulosa cells, is downregulated by hCG [9]. Andersen et al. analysed cortisol and cortisone in follicular fluid and found similar cortisol concentration in FF in NC-IVF and cIVF-follicles following ovulation induction with hCG [10].

Higher serum concentration of prolactin and cortisol in cIVF might affect the FF:S ratio. However, as we postulate that an increase of prolactin and cortisol in serum should result in similar relative increase of intrafollicular concentration, these differences in serum concentrations should have only minor effects on our study results.

It can therefore be assumed that the influence of the factors “conversion” and “increased serum concentrations” should have only limited effect on the FF:S ratio, allowing this ratio to be used as a indirect parameter for the analysis of the gonadotropin dependant blood-ovarian permeability of prolactin and cortisol.

As we have stated above, a gonadotropin induced increase of the blood-ovarian permeability should result in an increase of the FF:S ratio. However, we have found exactly the opposite i.e. a significant decrease of the ratio. Even many other factors might affect intrafollicular concentrations and thereby the FF:S ratio, we can most certainly conclude that gonadotropins did not increase the blood-follicular transportation capacity, as we would otherwise have observed an increase in the FF:S ratio in the cIVF group.

In summary, our model should allow the conclusion that gonadotropin stimulation does not increase the transportation of prolactin and cortisol into the follicle. This effect might possibly also apply to other hormones regulating ovarian function. It can therefore be assumed that the above mentioned negative effects of gonadotropins on follicular function are not due to an increase in blood-ovarian permeability, as it has been previously described in animal studies.

### Conclusions

The model of serum follicular fluid:serum ratio of hormones, produced outside the ovaries, did not reveal an gonadotropin induced increased follicular permeability. Therefore it can be assumed that the effect of gonadotropins on follicular endocrine function is not due to an increased permeability of extra-ovarian hormones.

## Competing interests

The study was supported by an unrestricted research grant from IBSA Institut Biochemique SA and MSD Merck Sharp & Dohme GmbH. The authors are clinically involved in low dose monofollicular stimulation and IVF-therapies, using gonadotropins from all gonadotropin distributers on the swiss market, including Institut Biochemique SA and MSD Merck Sharp & Dohme GmbH.

## Authors’ contributions

MvW: Conception and design, analysis and interpretation of data, drafting of the manuscript; SS: Analysis and interpretation of data; revision of the manuscript; ZK: Acquisition of data; revision of the manuscript; BW: Acquisition of data; revision of the manuscript; NAB: Analysis and interpretation of data; drafting of the figures, statistics. All authors read and approved the final manuscript.
